# Outcome of pulpotomy in permanent teeth with irreversible pulpitis: a systematic review and meta-analysis

**DOI:** 10.1038/s41598-022-20918-w

**Published:** 2022-11-16

**Authors:** Amber Ather, Biraj Patel, Jonathan A. L. Gelfond, Nikita B. Ruparel

**Affiliations:** 1 Department of Endodontics, Virginia Commonwealth University School of Dentistry, Richmond, VA 23298 USA; 2 Department of Endodontics, University of Texas Health Science Center at San Antonio, 7703 Floyd Curl Drive, San Antonio, TX 78229 USA; 3 Department of Population Health Sciences, UT Health San Antonio, 7703 Floyd Curl Drive, San Antonio, TX 78229 USA

**Keywords:** Data mining, Literature mining, Pulpitis

## Abstract

Treatment planning is key to clinical success. Permanent teeth diagnosed with “irreversible pulpitis” have long been implied to have an irreversibly damaged dental pulp that is beyond repair and warranting root canal treatment. However, newer clinical approaches such as pulpotomy, a minimally invasive and biologically based procedure have re-emerged to manage teeth with pulpitis. The primary aim of the study was to conduct a meta-analysis to comprehensively estimate the overall success rate of pulpotomy in permanent teeth with irreversible pulpitis as a result of carious pulp exposure. The secondary aim of the study was to investigate the effect of predictors such as symptoms, root apex development (closed versus open), and type of pulp capping material on the success rate of pulpotomy. Articles were searched using PubMed, Scopus, CENTRAL, and Web of Science databases, until January 2021. Outcomes were calculated by pooling the success rates with a random effect model. Comparison between the different subgroups was conducted using the z statistic test for proportion with significance set at alpha = 0.05. A total of 1,116 records were retrieved and 11 studies were included in the quantitative analysis. The pooled success rate for pulpotomy in teeth with irreversible pulpitis was 86% [95% CI: 0.76–0.92; *I*^2^ = 81.9%]. Additionally, prognostic indicators of success were evaluated. Stratification of teeth based on (1) symptoms demonstrated that teeth with symptomatic and asymptomatic irreversible pulpitis demonstrated success rate of 84% and 91% respectively, with no significant difference (p = 0.18) using z-score analysis; (2) open apex teeth demonstrated a significantly greater success rate (96%) compared to teeth with closed apex (83%) (p = 0.02), and (3) pulp capping materials demonstrated that Biodentine yielded significantly better success rates compared to Mineral Trioxide Aggregate (MTA), calcium hydroxide, and Calcium Enriched Mixture (CEM.) Collectively, this is the first meta-analytical study to determine the clinical outcome of pulpotomy for carious teeth with irreversible pulpitis and it’s predictors for success. Moreover, we identify the stage of root development and type of biomaterial as predictors for success of pulpotomy.

## Introduction

According to the Global Burden of Disease census, untreated dental caries in permanent teeth is the most prevalent health condition^1^. Failure to treat dental caries can lead to the spread of bacteria and their toxins into the dental pulp causing varying degrees of inflammation^2^. From a clinical point of view, pulpal inflammation is dichotomously classified as “reversible” or “irreversible” based on the presumed ability of the pulp to heal^3^. According to AAE Consensus Conference Recommended Diagnostic Terminology^3^, irreversible pulpitis is defined as “A clinical diagnosis based on subjective and objective findings indicating that the vital inflamed pulp is incapable of healing and that root canal treatment is indicated.”. As a result, pulp extirpation remains to be the mainstay treatment option for teeth diagnosed with irreversible pulpitis^3^. However, the validity of the diagnostic term “irreversible pulpitis” has come into question as an emerging body of evidence has shown that clinical diagnosis might not correlate with the histologic condition of the pulp^4^. It has been observed that even in advanced cases of inflammation with irreversible pulpitis, only isolated areas of pulp in the coronal aspect had bacterial invasion with micro abscesses, whereas the underlying pulp continued to be free of inflammation^4^. Moreover, upon removal of the coronal pulp in direct contact with bacterial insult, the pulp represents a tissue that demonstrates excellent regenerative potential^5^. Therefore, with advancements in the understanding of pulp biology along with the introduction of bioceramic materials, the concept of “vital pulp therapy” (VPT) has been revisited and there have been numerous reports of successful outcomes in teeth with carious pulp exposures or pulpitis^6–8^. Amongst VPT modalities, pulpotomy has been demonstrated to be a more predictable and successful intervention to manage teeth with carious pulp exposures compared to direct and indirect pulp capping procedures^9^.

Clinical data on the success of pulpotomy procedures in permanent teeth range from 82 to 100%^8–11^. Moreover, a recent systematic review demonstrated high success rates (> 90%) of coronal pulpotomy in mature teeth with irreversible pulpitis at a 1-year time point^12^. However, the contribution of risk factors (e.g. preoperative symptoms, stage of root development, and choice of pulp capping material) on the outcome of pulpotomy present a large gap in knowledge. Therefore, a lack of meta-analytical data using predictors of success or risk factors precludes the accurate prediction of clinical outcomes. Therefore, the *primary aim* of this comprehensive systematic review and meta-analysis was to evaluate the success rate of pulpotomy in carious teeth diagnosed with irreversible pulpitis at a minimum of 6 months post-treatment. The *secondary aim* of the study was to investigate the effect of predictors such as symptoms, root apex development (closed versus open), and type of pulp capping material on the success rate of pulpotomy. To our knowledge, this is the first study evaluating the aforementioned variables on the outcome of pulpotomy for permanent teeth with irreversible pulpitis as a result of carious pulp exposure.

## Materials and methods

This systematic review was conducted in accordance with the Preferred Reporting Items for Systematic Reviews and Meta-Analyses (PRISMA) guidelines^13^.

### Focused question

This study adhered to the PIO framework to address the following clinical question: “What is the success rate of pulpotomy in human permanent teeth with a diagnosis of irreversible pulpitis?”, wherein the population (P) is “human permanent teeth with irreversible pulpitis as a result of carious pulp exposure”; intervention (I) is “pulpotomy”; outcome of interest (O) is “success rate based on clinical and radiographic criteria”.

### Inclusion criteria

1. Original clinical data namely, clinical trials, prospective and retrospective observational studies reporting on pulpotomy in human permanent teeth
2. Teeth with carious pulp exposure and signs and symptoms suggestive of irreversible pulpitis
3. Studies that had appropriate use of both clinical and radiographic criteria to judge and report success rate. Clinical success was defined as absence of clinical manifestations such as pain on percussion/palpation and spontaneous pain, and devoid of need for further root canal treatment. Radiographic success was defined as maintenance of normal periapical tissues (in case of absence of pre-operative lesions) or complete or continued healing of periapical tissues (in case of presence of pre-operative lesions).
4. Minimum follow up period of 6 months
5. English language only

### Exclusion criteria

1. Case reports, case series, or review papers
2. Studies reporting on pulpotomy in deciduous teeth
3. Studies reporting on pulpotomy in teeth with trauma
4. Studies using lasers in pulpotomy
5. Studies not reporting on success rate or where raw data was not available to calculate success rate
6. Studies reporting on other vital pulp therapy procedures such as direct or indirect pulp capping

### Search strategy

Electronic search was conducted by two independent reviewers (A.A. and B.P.) using databases such as PubMed, Scopus, CENTRAL, and Web of Science. Keywords pertinent to the topic in question were used in various combinations using Boolean operators to extract the relevant studies. Search strategy used on PubMed was adapted for other databases (Supplementary Table S1). Articles published between January 1960 and January 2021 were included in the screening process. In addition, the bibliography of included articles and review papers were screened to find any missing studies. The identified studies were then exported into Mendeley reference manager (Mendeley Desktop, version 1.17.11; Mendeley Ltd., George Mason University, Fairfax, VA) and any duplicate articles were removed.

### Study selection process

A two-phase search strategy was followed, wherein two independent reviewers (A.A. and B.P.) screened the titles and abstract of all extracted articles in the first phase and subjected them to the inclusion/exclusion criteria to perform preliminary elimination of ineligible studies. In the second stage, full text of the articles was retrieved and evaluated for inclusion in the meta-analysis. Any duplication of data presented in studies was noted and eliminated for statistical analysis. Any disagreements in the search and screening process between the reviewers was resolved by discussion.

### Data extraction

Two independent reviewers (A.A., B.P.) performed data extraction from eligible studies using customized data retrieval forms on Microsoft Excel (Microsoft Office; Microsoft, Redmond, WA). Extracted data included author/year, study design, sample size, diagnosis, root apex development (open or closed), capping material, pulpotomy type (partial or full), proportion of successful cases, follow up period, and recall rate. In cases of duplication of data amongst different studies, study with the maximum follow up period was included for cumulative analysis. In studies reporting on mixed clinical scenarios, efforts were made to extract raw data pertinent to the inclusion criteria and for evaluating secondary objectives of the meta-analysis. In case of missing information, authors of the reports were contacted via email to gather further details.

### Quality evaluation of included studies

The risk of bias of included studies was assessed based on the study design. The non-randomized studies were assessed by modified Down and Black’s checklist^14^. This checklist is based on 27 questions divided amongst 5 different sections: reporting, external validity, internal validity, confounding and selection bias, and power of the study. The total score ranging from 0 to 28 was assigned to each study^14^. Randomized clinical trials were assessed using the Cochrane Risk of Bias 2 (RoB 2) tool^15,16^. This tool has fixed domains with signaling questions which can derive information about key aspects of clinical trials relevant to risk of bias. Studies can be rated as having “low”, “some concerns” or “high” risk of bias. The assessments were performed by two examiners (A.A., B.P.). Any discrepancy in quality evaluation was resolved via discussion.

### Data synthesis and statistical analysis

The primary outcome formulated after data collection was the overall success rate of pulpotomy in cariously involved permanent teeth with irreversible pulpitis. The goal of the subgroup analysis was to assess secondary outcomes which included comparison of success rate under different clinical contexts. This included estimation of outcome based on symptoms (symptomatic versus asymptomatic), stage of root apex development (closed versus open), and pulp capping material used.

All the outcomes were calculated by pooling the success rates with a random effect model^17^. Heterogeneity across the studies was assessed using Cochrane *I*^2^ statistic (*I*^2^ value of > 60% was considered as significant heterogeneity)^18^. Comparison between the different subgroups was conducted using the z statistic test for proportion with significance set at alpha = 0.05. Publication bias was assessed using funnel plot and performing the Egger’s test^19^. In case of publication bias, trim and fill method was used to impute missing studies and adjust the effect of bias^20^. Comprehensive Meta-Analysis (Version 3; Biostat, Englewood, NJ) software package was used to perform the meta-analyses and publication-bias analyses.

### Grading of evidence

The Grading of Recommendations, Assessment, Development and Evaluation (GRADEpro GDT: GRADEpro Guideline Development Tool; McMaster University, Hamilton, ON, Canada) was used to assess the quality of evidence^21^. Two reviewers (A.A., B.P.) performed the quality analysis based on the following domains: risk of bias, inconsistency of results, indirectness of evidence, imprecision, and publication bias^21^. In cases of disagreement, a consensus was reached by discussing with the third reviewer (N.B.R.).

## Results

### Selected studies

**Figure 1:**
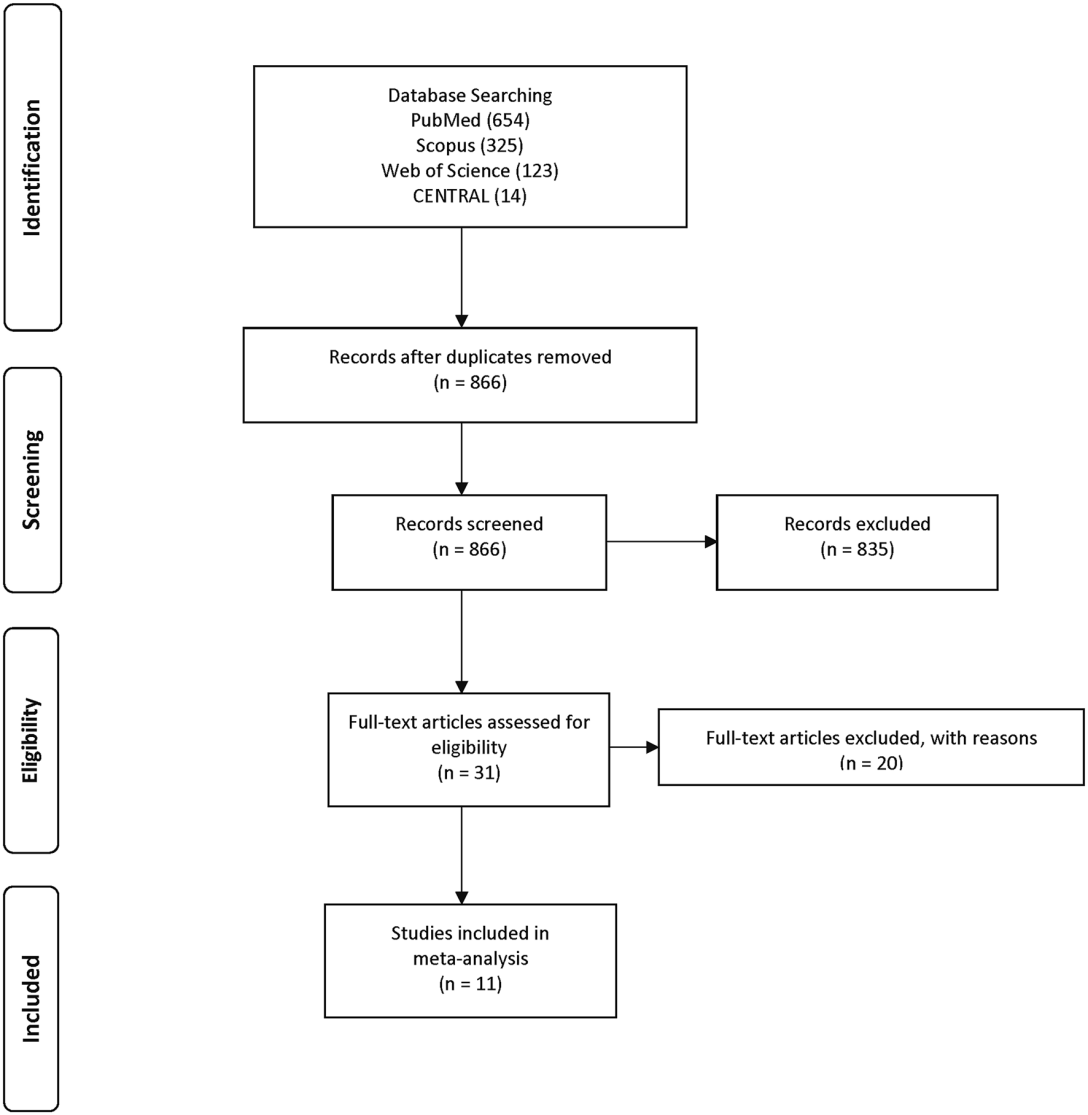
Flowchart for study selection process.

The study selection process is outlined in Fig. 1. The initial search yielded 1,116 records, out of which 866 were screened based on title and abstract. After exclusion of 835 articles, 31 full text articles were retrieved and screened for eligibility. 11 studies met the inclusion criteria^7,22–31^, and relevant data was extracted for performing the meta-analysis. Reasons for exclusion of each full text article is listed in Supplementary Table S2. Overall, there were 5 randomized control trials^22,25,27–29^, 5 prospective clinical studies^23,24,26,30,31^ and one retrospective study^7^. The details and characteristics of included studies^7,22–31^ are outlined in Table 1.

**Table 1:** Characteristics of the included studies in the systematic review.

Author, year	Design	Sample size	Root apex development	Pulpal diagnosis	Capping material	Type of pulpotomy	Success n/N	Follow up (months)	Recall rate (%)
Uesrichai, 2019^22^	Randomized clinical trial	69	Mixed	SIP	Proroot MTA, BD	Partial	34/37 (MTA), 26/30 (BD)	32 ± 17.9	97
Taha, 2018^23^	Prospective	20	Mixed	SIP	Biodentine	Full	19/20	12	100
Taha, 2018^24^	Prospective	63	Closed	SIP	Biodentine	Full	58/59	12	93.6
Taha, 2017^25^	Randomized clinical trial	50	Closed	SIP	Proroot MTA, Ca(OH)2	Partial	22/26 (MTA), 10/23 (Ca(OH)2)	12–24	90
Linsuwanont, 2017^7^	Retrospective	66	Mixed	SIP = 25	ProRoot MTA	Full	21/25	8–62	82
Qudeimat, 2017^26^	Prospective	23	Mixed	IP (SIP 21)	ProRoot MTA	Full	23/23 (IP), 21/21 (SIP), 2/2 (AIP)	18.9–73.6	100
Asgary, 2017^27^	Randomized clinical trial	412	Closed	SIP	ProRoot MTA, CEM	Full	116/154 (MTA), 107/150 (CEM)	60	74
Kumar, 2016^28^	Randomized clinical trial	54	Closed	SIP	ProRoot MTA, Ca(OH)2	Full	6/16 (Ca(OH)2), 8/18 (MTA)	12	90
Nosrat, 2013^29^	Randomized clinical trial	34	Open	SIP	ProRoot MTA, CEM	Full	17/17 MTA, 17/17 CEM	12	NR
Caliskan, 1995^30^	Prospective	26	Closed	AIP	Ca(OH)2	Full	24/26	16–72	100
Caliskan, 1993^31^	Prospective	24	Closed	AIP	Ca(OH)2	Full	22/24	12–48	100

IP—irreversible pulpitis, SIP—symptomatic irreversible pulpitis, AIP—asymptomatic irreversible pulpitis, MTA—mineral trioxide aggregate, BD—biodentine, CEM—calcium enriched mixture, Ca(OH)^2^—calcium hydroxide, n—successful outcomes, N—sample size, NR—not reported.

### Quality assessment

After analysis using modified Downs and black’s checklist, the quality assessment of non-randomized studies^7,23,24,26,30,31^ revealed all studies to be of fair quality (Supplementary Table S3). For randomized control trials assessed with the RoB 2 tool, 2 studies had “some concerns” with risk of bias^27,28^, whereas 3 studies had “low” risk of bias^22,25,29^ (Supplementary Table S4).

### Primary analysis

#### Pooled success rate of pulpotomy in IP

**Figure 2:**
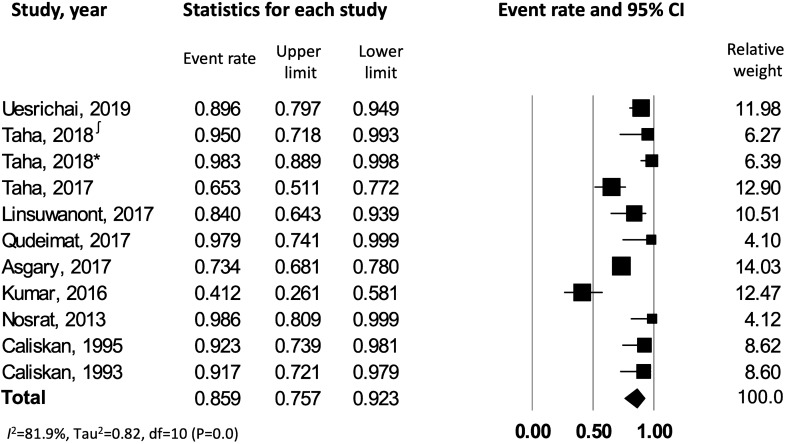
Success rate of pulpotomy in carious teeth with irreversible pulpitis. ∫ corresponds to reference number 23, whereas * corresponds to reference number 24.

After combining the results from 11 selected studies^7,22–31^ with random effects model, the overall pooled success rate of pulpotomy in carious permanent teeth with irreversible pulpitis (asymptomatic and symptomatic) was 86% [CI: 0.76–0.92] with significant heterogeneity across the studies (*I*^*2*^ = 81.9%) (Fig. 2).

### Subgroup analysis

#### Pooled success rate of pulpotomy in teeth diagnosed with symptomatic and asymptomatic irreversible pulpitis

**Figure 3:**
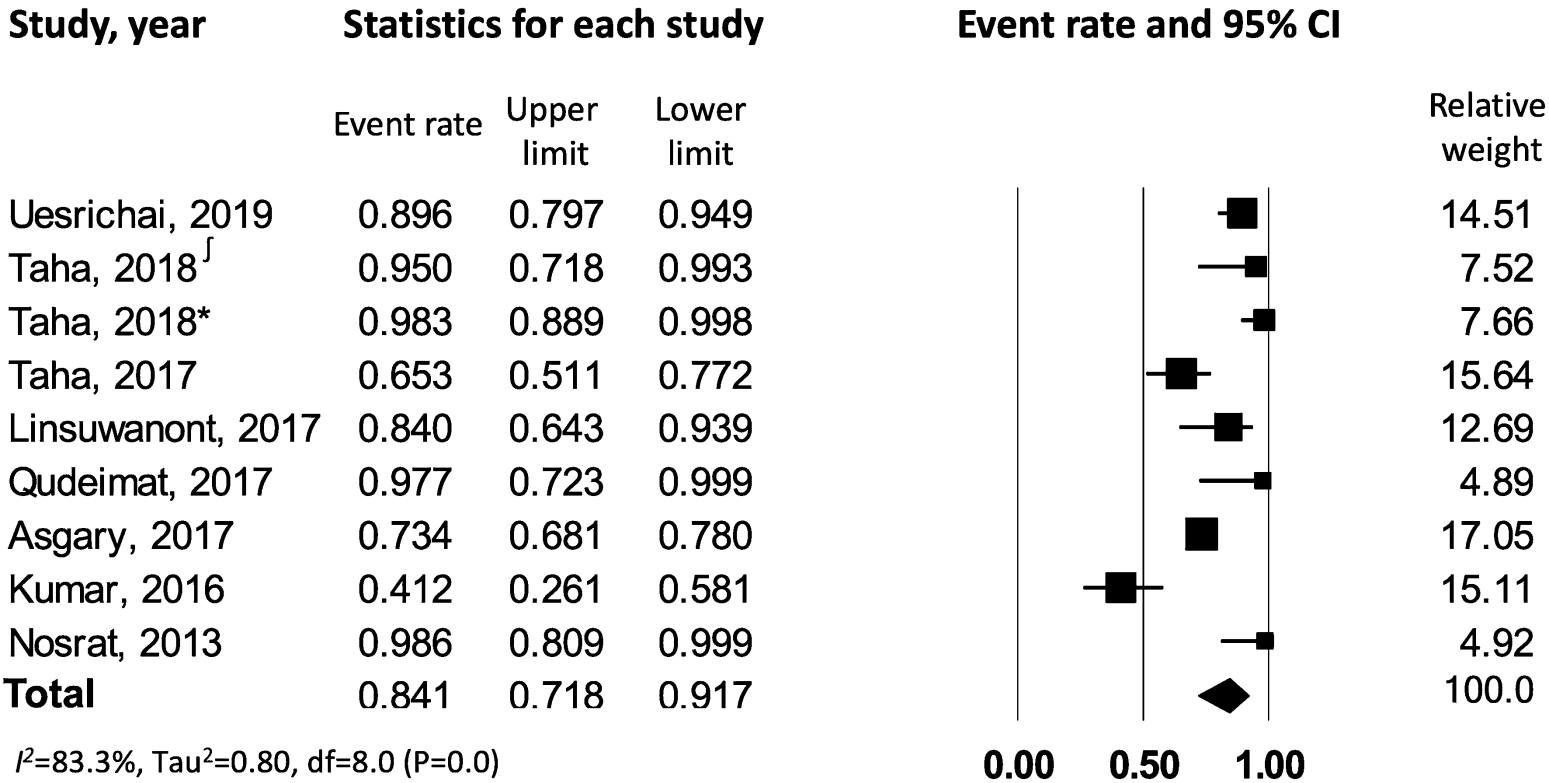
Success rate of pulpotomy in carious teeth with symptomatic irreversible pulpitis. ∫ corresponds to reference number 23, whereas * corresponds to reference number 24.

**Figure 4:**
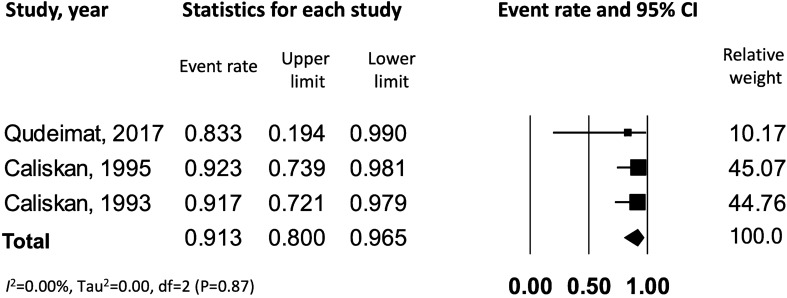
Success rate of pulpotomy in carious teeth with asymptomatic irreversible pulpitis.

The data were further analyzed to assess the outcome of pulpotomy in carious teeth with *symptomatic versus asymptomatic irreversible pulpitis*. This resulted in 9 studies^7,22–29^ meeting the inclusion criteria of symptomatic irreversible pulpitis, which were combined to yield a success rate of 84% [CI: 0.72–0.92], with a significant heterogeneity across the studies (*I*^*2*^ = 83.3%) (Fig. 3). Data from 3 studies^26,30,31^ reporting on asymptomatic irreversible pulpitis were combined to yield a success rate of 91.3% [CI: 0.80–0.96.5], with no observed statistical heterogeneity across the studies (*I*^*2*^ = 0.00%) (Fig. 4). On comparing symptomatic versus asymptomatic teeth, there was no significant difference observed in terms of pulpotomy success rate (z value for proportion: −1.34; p = 0.18).

#### Pooled success rate of pulpotomy for irreversible pulpitis in teeth with closed versus open apex

**Figure 5:**
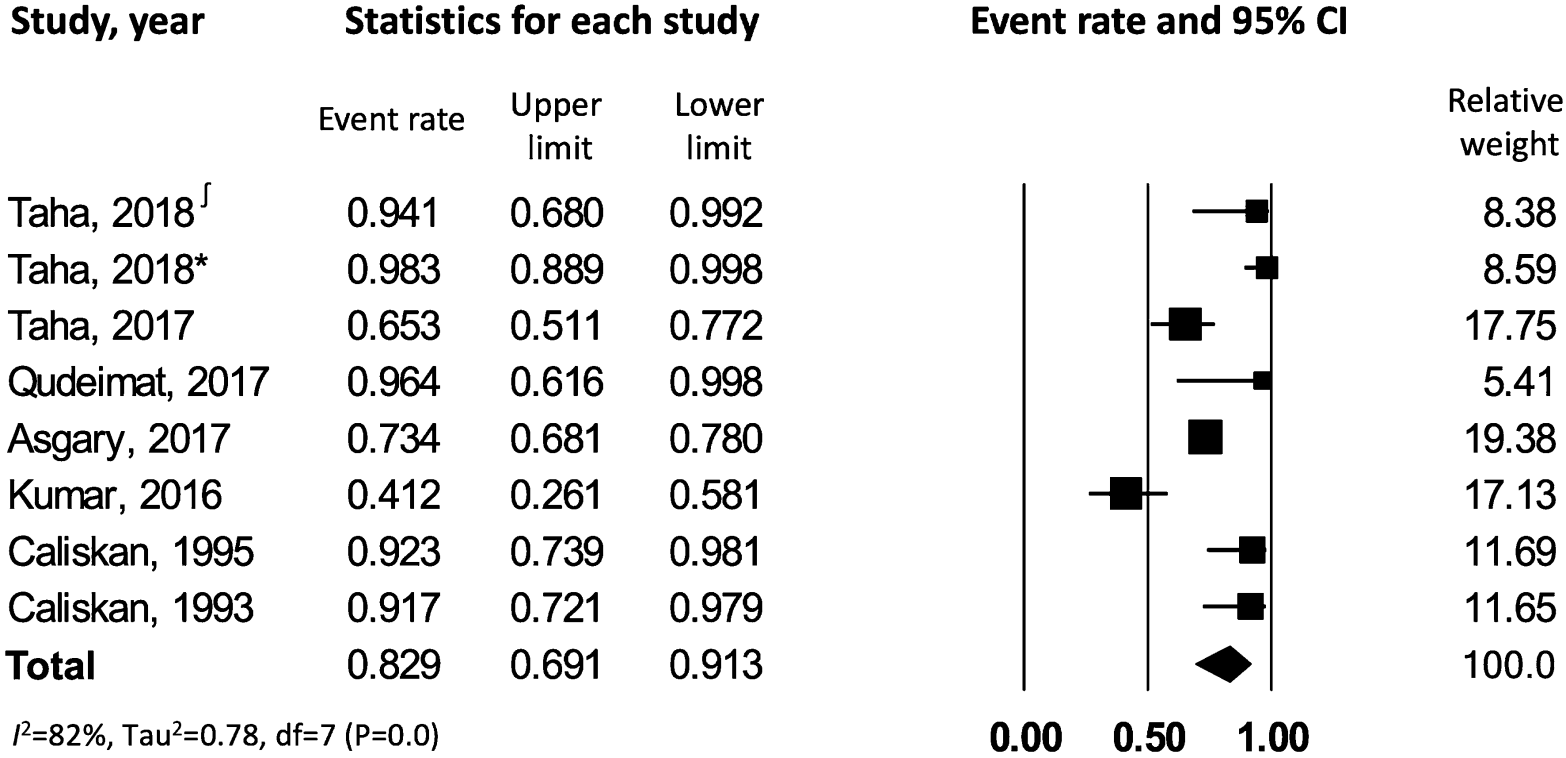
Success rate of pulpotomy in carious closed apex teeth with irreversible pulpitis. ∫ corresponds to reference number 23, whereas * corresponds to reference number 24.

**Figure 6:**
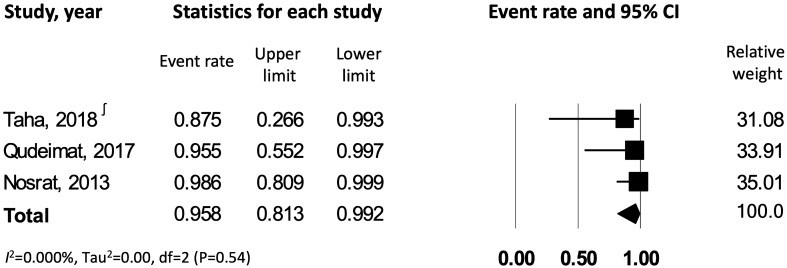
Success rate of pulpotomy in carious open apex teeth with irreversible pulpitis. ∫ corresponds to reference number 23.

The data were stratified to identify the effect of closed versus open root apex on success rate of pulpotomy in carious teeth with irreversible pulpitis. 8 studies^23–28,30,31^ were identified with pulpotomy performed in teeth with complete root development (closed apex); after pooling these studies with random effects model, the overall success rate of pulpotomy in teeth with irreversible pulpitis and closed apex was 83% [CI: 0.69–0.91], with significant heterogeneity amongst the studies (*I*^*2*^ = 82%) (Fig. 5). In comparison to teeth with closed apices, there were only 3 studies^24,26,29^ reporting on pulpotomy in teeth with incomplete root development (open apex), which demonstrated a cumulative success rate of 95.8% [CI: 0.81–0.99], with no evidence of heterogeneity across the studies (*I*^*2*^ = 0.00%) (Fig. 6). Open apex teeth demonstrated a significantly higher success rate when compared to teeth with closed apex (z value for proportion: 2.33; p = 0.02).

#### Pooled success rate of pulpotomy for irreversible pulpitis in teeth restored with various pulp capping materials

The effect of pulp capping materials on success rate were compared with indirect comparisons between studies^7,22–31^. Biodentine demonstrated a statistically significant higher success rate when compared to MTA, Calcium Enriched Mixture (CEM), and calcium hydroxide. MTA yielded a significantly higher success rate when compared to calcium hydroxide; however, it was not significantly superior to CEM. Similarly, there was no significant difference observed between CEM and calcium hydroxide (Supplementary Table S5).

Visual analysis of the funnel plot and Egger’s regression test (p < 0.04) indicated potential publication bias (Supplementary Table S6). Using the “trim and fill” method, 4 studies were imputed to the left of the funnel plot, which yielded a point estimate of 0.786 (95% CI: 0.66–0.87). This point estimate, subsequent to adjusting for potential publication bias, indicates a 78.6% success rate of pulpotomy in teeth with irreversible pulpitis.

### Grading of evidence

The certainty of evidence was graded as “very low” (Table 2), which means that the true effect is probably markedly different from the estimated effect of 86% pooled success of pulpotomy in carious teeth with irreversible pulpitis.

**Table 2:** Grading of recommendations assessment, development and evaluation approach to grade quality of evidence.

No. of studies	Certainty assessment						Effect			Certainty
	Study design	Risk of bias	Inconsistency	Indirectness	Imprecision	Other considerations	No. of events	№ of individuals	Event Rate (95% CI)	
5	Randomized trials	Serious	Very serious	Not serious	Serious	Publication bias strongly suspected strong association	363	488	74.3 per 100 (0.56 to 0.86)	⨁◯◯◯ Very low
6	Observational studies	Serious	Very serious	Not serious	Serious	Publication bias strongly suspected strong association	167	177	92.6 per 100 (0.86 to 0.96)	⨁◯◯◯ Very low

There are some concerns with the randomization process and in the measurement of outcome.

There are serious concerns with the inconsistency as evidenced by the significant heterogeneity amongst studies.

Total number of subjects with successful outcome is less than 400.

There are some concerns with controlling the confounding factors.

Overall effect size is too large compared to the sample size.

## Discussion

Pulpotomy, an often-overlooked vital pulp therapy procedure, has now re-emerged as a minimally invasive, biologically based treatment option for teeth diagnosed with pulpitis and involves partial/ complete removal of coronal pulp tissue, following which a biocompatible material is placed onto the pulp tissue to promote healing^6^. Specifically, it was seldom used as a definitive treatment modality in teeth with symptomatic irreversible pulpitis based on diagnostic modalities that predominantly serve to assess neuronal function as a measure of pulpal health^32^. However, with recent advances in newer biocompatible, anti-inflammatory and osteo-inductive biomaterials, the face of VPT has evolved^32^. Therefore, we conducted a systematic review and meta-analysis to assess the outcome of pulpotomy in teeth with irreversible pulpitis using available clinical data. With very low certainty, our study demonstrates pulpotomy to be a successful intervention for carious teeth with irreversible pulpitis with a favorable outcome of 86%. Our results substantiate the findings by Cushley et al., wherein successful outcomes were demonstrated in 88% of cases at 3-years^12^. Additionally, the present study computed success rates using meta-analytical methods to include relative weight of each study and allows for random variation in the success rate among studies. The present study also performed a comprehensive assessment of risk factors on the outcome of clinical success. Our results of this subset analyses evaluated the following variables/risk factors: teeth diagnosed with symptomatic versus asymptomatic irreversible pulpitis, teeth with mature (closed) versus immature (open) root apex, and choice of pulp capping material used.

Based on the clinical presentation and diagnostic testing, irreversible pulpitis is classified as symptomatic or asymptomatic^3^. In symptomatic cases, pulpotomy has traditionally been used as an emergency procedure to relieve pain^33^. In addition, presence of acute preoperative symptoms is typically regarded as a negative outcome predictor for long-term success of VPT^34^, thereby precluding application of pulpotomy as a definitive treatment modality. However, in this meta-analysis, we demonstrate a success rate of 84% of pulpotomy in teeth with SIP, which was not significantly different from the outcome observed in teeth with asymptomatic irreversible pulpitis. Studies that included teeth with symptomatic irreversible pulpitis defined these cases as teeth with either spontaneous pain and/or pain exacerbated by cold stimuli with prolonged episodes of pain even after the thermal stimulus had been removed^7,22–27^.This favorable outcome in symptomatic irreversible pulpitis can be attributed to the fact that pulpotomy procedures regulate immune responses and can reduce levels of pro-inflammatory cytokines within the dental pulp^35^. In addition, histologic studies have demonstrated that in teeth with irreversible pulpitis, the damage and inflammation was mostly confined to only a portion of the coronal pulp, with the rest of the coronal and radicular pulp being intact and healthy^4^. Furthermore, anti-inflammatory properties of new generation tricalcium silicate materials promote reversal of residual inflammation and maintenance of a healthy pulp tissue thereafter^36^. Collectively, these above mentioned reasons suggest that the removal of the coronally inflamed pulp might be sufficient to maintain viability and health of the radicular pulp, making pulpotomy an effective emergency procedure as well as a definitive treatment modality in this subset of patient population.

Traditional VPT procedures were aimed at promoting continued root development (apexogenesis)^37^. Therefore, clinically, teeth with fully formed apices (closed apex) were excluded from VPT case selection. However, with the growing knowledge about the repair potential of the dental pulp^38^, VPT such as pulpotomy protocols have been introduced to treat teeth with closed apex with a diagnosis of irreversible pulpitis^12^. The results from this meta-analysis demonstrate that pulpotomy in teeth with closed apex yields a cumulative success rate of 83%. In contrast, teeth with open apex demonstrated a significantly favorable outcome. This can be attributed to the increased vascularity and cellularity of pulp in immature teeth^39^. In addition, aging of dental pulp is associated with reduced regenerative potential of dental stem cells^40^. However, with a success rate of 83% in mature teeth with irreversible pulpitis, pulpotomy should be considered a viable and definitive treatment approach in these cases. These results are in accordance with the findings demonstrated by Tan et al.^34^ and Kunert et al.^41^, wherein favorable success rates were reported for both young immature as well as mature teeth.

Pulp capping agents can affect the outcome of pulpotomy procedures^37^. Traditionally, calcium hydroxide has been the most popular pulp capping agent, owing to its antimicrobial nature and the capability to form a hard tissue barrier; however, issues such as high solubility, lower mechanical resistance and presence of tunnel defects in the mineralized barrier were reported as concerns especially for vital pulp therapy procedures^42^. Tricalcium silicate-based materials such as MTA and alike have now become the material of choice for pulpotomy because of several added advantages such as biocompatibility, reduced microleakage, ability to induce a thicker dentinal bridge with fewer defects and ability to release growth factors from dentin^36,41^. MTA as a pulp capping material has few disadvantages such as its potential to discolor tooth and high solubility owing to the slow setting reaction^43^. Other bioceramic materials such as Biodentine and CEM have been introduced to overcome these shortcomings of MTA. In contrast to MTA, Biodentine has been demonstrated to cause lesser tooth discoloration^44^. CEM is another bioceramic material introduced in 2006 with properties similar to MTA, however with better physical characteristics and shorter setting time^45^. We therefore wanted to evaluate the success rate of pulpotomy in teeth with irreversible pulpitis based on the types of biomaterial used. In our meta-analysis, Biodentine demonstrated to be superior to other pulp capping materials, in terms of success rate for pulpotomy. This finding might be attributed to the ability of biodentine to cause a greater release of calcium ions and bioactive growth factors^46^. MTA was found to be superior to calcium hydroxide, which corroborates the results from study done by Li et al.^32^. Interestingly, CEM and calcium hydroxide demonstrated similar success rates. Collectively, these data suggest that use of bioceramic pulp capping materials such as MTA and Biodentine can lead to more favorable clinical outcomes in teeth with irreversible pulpitis. Our findings substantiate the results of a recent systematic review by Sabeti et al., wherein a 93% success rate with VPT procedures utilizing bioceramic pulp capping materials was obtained^47^.

From a clinical and research point of view, there are several factors in vital pulp therapy, which still need to be standardized. There is a lack of agreement regarding the indications for pulpotomy procedures^48^. This is partly attributable to the diagnostic ambiguity of our current pulp testing methods to establish the true inflammatory status of pulp^38^. As for the choice of type of pulpotomy, there is some evidence that full pulpotomy is more successful than partial pulpotomy especially in cases with irreversible pulpitis^9^. Interestingly, most of the included studies in our meta-analysis employed full coronal pulpotomy^7,23,24,26–31^, therefore a subset analysis based on type of pulpotomy was not performed. Traditionally, a minimum of 3–6 months follow up time has been established to determine the prognosis of a vital pulp therapy procedure^49–51^. This follow up time point interval is critical, as majority of the early failures present during this period and it has been demonstrated that pain or symptoms during the first 3 months after VPT are associated with poor outcomes^49^. Accordingly, studies with at least a 6-month follow up period were included in this meta-analysis^7,22–31^. The included studies had intra- and inter- study variation in patient recruitment which led to a wide range of follow up periods. In addition, only select studies reported on long term outcomes (> 2 years) of VPT procedures^7,22,26,27,30^. As a result, data was not analyzed based on specific follow up time intervals. Regarding the coronal seal after pulpotomy, all the included studies had either a permanent restoration placed immediately after the procedure or a glass ionomer liner placement followed by a temporary restoration. None of the studies delayed the placement of final restoration beyond a month’s duration^7,22–31^.

Our study has some limitations and should be interpreted with caution. One of the limitations was the considerable heterogeneity across the included studies^7,22–31^. This heterogeneity could be attributed to multiple factors such as variation in study design, patient selection, type of pulpotomy, choice of biomaterial, and follow up period, to mention a few. However, we explored the reasons for heterogeneity in our meta-analysis by performing subgroup analysis. No heterogeneity was reported when analysis was restricted to immature teeth and asymptomatic teeth subgroups. None of the other studied variables could resolve the observed heterogeneity in the meta-analysis. Another key limitation to note is the inconsistency of the included studies with the use of sensibility testing at follow-up periods^7,22–31^. Only 4 studies utilized sensibility testing as a criterion at follow-up appointments to evaluate success^22,25,30,31^. Therefore, while radiographic assessment satisfies one of the criteria for disease-centered outcome, inclusion of vitality of treated teeth would define a true disease-centered outcome and greatly increase the pragmatic significance to both, clinicians and patients.

There are several confounding moderators, which need to be taken into account and should be consistently reported in future vital pulp therapy research. These include patient and tooth specific factors (such as age, gender, tooth type), operator factors (investigator specialty and experience), technical factors (type of pulpotomy, details of hemostatic agent used and time for hemostasis, choice of biomaterial and permanent restoration) and data from recall appointments. Having these factors reported in future studies will help other researchers and clinicians to understand the outcomes better and will also improve the applicability and generalizability of the results.

## Conclusion

The success rate obtained in this meta-analysis adds to the emerging body of evidence supporting the role of pulpotomy as a definitive treatment modality in carious teeth with irreversible pulpitis. Given the potential of pulpotomy in managing teeth with pulpitis and global rise in minimally invasive dentistry, there is a tremendous need for conducting well-designed randomized clinical trials on this understudied topic.

## Supplementary Information

Supplementary Information 1.

Supplementary Information 2.

## Data Availability

The datasets generated during and/or analyzed during the current study are available from the corresponding author on reasonable request.
