# Supplementary material for: Outcome of pulpotomy in permanent teeth with irreversible pulpitis: a systematic review and meta-analysis
Source: Sci Rep. 2022 Nov 16;12:19664. doi: 10.1038/s41598-022-20918-w (PMC9669040; doi:10.1038/s41598-022-20918-w)

**Outcome of Pulpotomy in Permanent Teeth with Irreversible Pulpitis: A Systematic Review and Meta-Analysis**

Amber Ather, BDS, DDS, MS^1^, Biraj Patel BDS, MS^2^, Jonathan A. L. Gelfond, MD, PhD^3^, Nikita B. Ruparel, MS, DDS, PhD^2^*

^1^ Department of Endodontics, Virginia Commonwealth University School of Dentistry, Richmond, Virginia 23298

^2^ Department of Endodontics, University of Texas Health Science Center at San Antonio, San Antonio, Texas 78229

^3^ Department of Population Health Sciences, UT Health San Antonio, 7703 Floyd Curl Drive, San Antonio, TX 78229

**Running title**: Pulpotomy in teeth with irreversible pulpitis

***Corresponding Author:**

Nikita Ruparel

Department of Endodontics, University of Texas Health Science Center at San Antonio, 7703 Floyd Curl Dr, San Antonio, Texas 78229

Phone: (210)-567-3396; Fax: not available; E-mail: [ruparel@uthscsa.edu](mailto:ruparel@uthscsa.edu)

**CONFLICT OF INTEREST**

The authors declare no conflict of interest.

**Supplementary S1. Literature search strategy**

Advanced search strategy used on PubMed. Search strategy was then adapted for other databases. The search was last done in January 2021.

| **Search number** | **Query** | **Sort By** | **Filters** | **Search Details** | **Results** | **Time** |
| --- | --- | --- | --- | --- | --- | --- |
| **4** | ((((((vital pulp) OR (vital pulp therapy)) OR (pulp capping)) OR (pulpotomy[MeSH Terms])) OR (agent, pulp capping[MeSH Terms])) AND (((((outcome) OR (survival)) OR (success)) OR (prognosis)) OR (healing))) AND (((((dental caries) OR (caries)) OR (cariously)) OR (pulpitis)) OR (pulpal inflammation)) |  |  | ((("vital signs"[MeSH Terms] OR ("vital"[All Fields] AND "signs"[All Fields]) OR "vital signs"[All Fields] OR "vital"[All Fields] OR "vitally"[All Fields] OR "vitals"[All Fields]) AND ("dental pulp"[MeSH Terms] OR ("dental"[All Fields] AND "pulp"[All Fields]) OR "dental pulp"[All Fields] OR "pulp"[All Fields])) OR (("vital signs"[MeSH Terms] OR ("vital"[All Fields] AND "signs"[All Fields]) OR "vital signs"[All Fields] OR "vital"[All Fields] OR "vitally"[All Fields] OR "vitals"[All Fields]) AND ("dental pulp"[MeSH Terms] OR ("dental"[All Fields] AND "pulp"[All Fields]) OR "dental pulp"[All Fields] OR "pulp"[All Fields]) AND ("therapeutics"[MeSH Terms] OR "therapeutics"[All Fields] OR "therapies"[All Fields] OR "therapy"[MeSH Subheading] OR "therapy"[All Fields] OR "therapy s"[All Fields] OR "therapys"[All Fields])) OR ("dental pulp capping"[MeSH Terms] OR ("dental"[All Fields] AND "pulp"[All Fields] AND "capping"[All Fields]) OR "dental pulp capping"[All Fields] OR ("pulp"[All Fields] AND "capping"[All Fields]) OR "pulp capping"[All Fields]) OR "pulpotomy"[MeSH Terms] OR "pulp capping and pulpectomy agents"[MeSH Terms]) AND ("outcome"[All Fields] OR "outcomes"[All Fields] OR ("mortality"[MeSH Subheading] OR "mortality"[All Fields] OR "survival"[All Fields] OR "survival"[MeSH Terms] OR "survivability"[All Fields] OR "survivable"[All Fields] OR "survivals"[All Fields] OR "survive"[All Fields] OR "survived"[All Fields] OR "survives"[All Fields] OR "surviving"[All Fields]) OR ("success"[All Fields] OR "successes"[All Fields] OR "successful"[All Fields]) OR ("prognosis"[MeSH Terms] OR "prognosis"[All Fields] OR "prognoses"[All Fields]) OR ("healed"[All Fields] OR "wound healing"[MeSH Terms] OR ("wound"[All Fields] AND "healing"[All Fields]) OR "wound healing"[All Fields] OR "healing"[All Fields] OR "healings"[All Fields] OR "heals"[All Fields])) AND ("dental caries"[MeSH Terms] OR ("dental"[All Fields] AND "caries"[All Fields]) OR "dental caries"[All Fields] OR ("carie"[All Fields] OR "dental caries"[MeSH Terms] OR ("dental"[All Fields] AND "caries"[All Fields]) OR "dental caries"[All Fields] OR "caries"[All Fields]) OR "cariously"[All Fields] OR ("pulpitis"[MeSH Terms] OR "pulpitis"[All Fields] OR "pulpitides"[All Fields]) OR (("pulpal"[All Fields] OR "pulpally"[All Fields]) AND ("inflammation"[MeSH Terms] OR "inflammation"[All Fields] OR "inflammations"[All Fields] OR "inflammation s"[All Fields]))) | 654 | 00:08:12 |
| **3** | ((((dental caries) OR (caries)) OR (cariously)) OR (pulpitis)) OR (pulpal inflammation) |  |  | "dental caries"[MeSH Terms] OR ("dental"[All Fields] AND "caries"[All Fields]) OR "dental caries"[All Fields] OR ("carie"[All Fields] OR "dental caries"[MeSH Terms] OR ("dental"[All Fields] AND "caries"[All Fields]) OR "dental caries"[All Fields] OR "caries"[All Fields]) OR "cariously"[All Fields] OR ("pulpitis"[MeSH Terms] OR "pulpitis"[All Fields] OR "pulpitides"[All Fields]) OR (("pulpal"[All Fields] OR "pulpally"[All Fields]) AND ("inflammation"[MeSH Terms] OR "inflammation"[All Fields] OR "inflammations"[All Fields] OR "inflammation s"[All Fields])) | 68,297 | 00:07:17 |
| **2** | ((((outcome) OR (survival)) OR (success)) OR (prognosis)) OR (healing) |  |  | "outcome"[All Fields] OR "outcomes"[All Fields] OR ("mortality"[MeSH Subheading] OR "mortality"[All Fields] OR "survival"[All Fields] OR "survival"[MeSH Terms] OR "survivability"[All Fields] OR "survivable"[All Fields] OR "survivals"[All Fields] OR "survive"[All Fields] OR "survived"[All Fields] OR "survives"[All Fields] OR "surviving"[All Fields]) OR ("success"[All Fields] OR "successes"[All Fields] OR "successful"[All Fields]) OR ("prognosis"[MeSH Terms] OR "prognosis"[All Fields] OR "prognoses"[All Fields]) OR ("healed"[All Fields] OR "wound healing"[MeSH Terms] OR ("wound"[All Fields] AND "healing"[All Fields]) OR "wound healing"[All Fields] OR "healing"[All Fields] OR "healings"[All Fields] OR "heals"[All Fields]) | 5,640,856 | 00:02:39 |
| **1** | ((((vital pulp) OR (vital pulp therapy)) OR (pulp capping)) OR (pulpotomy[MeSH Terms])) OR (agent, pulp capping[MeSH Terms]) |  |  | (("vital signs"[MeSH Terms] OR ("vital"[All Fields] AND "signs"[All Fields]) OR "vital signs"[All Fields] OR "vital"[All Fields] OR "vitally"[All Fields] OR "vitals"[All Fields]) AND ("dental pulp"[MeSH Terms] OR ("dental"[All Fields] AND "pulp"[All Fields]) OR "dental pulp"[All Fields] OR "pulp"[All Fields])) OR (("vital signs"[MeSH Terms] OR ("vital"[All Fields] AND "signs"[All Fields]) OR "vital signs"[All Fields] OR "vital"[All Fields] OR "vitally"[All Fields] OR "vitals"[All Fields]) AND ("dental pulp"[MeSH Terms] OR ("dental"[All Fields] AND "pulp"[All Fields]) OR "dental pulp"[All Fields] OR "pulp"[All Fields]) AND ("therapeutics"[MeSH Terms] OR "therapeutics"[All Fields] OR "therapies"[All Fields] OR "therapy"[MeSH Subheading] OR "therapy"[All Fields] OR "therapy s"[All Fields] OR "therapys"[All Fields])) OR ("dental pulp capping"[MeSH Terms] OR ("dental"[All Fields] AND "pulp"[All Fields] AND "capping"[All Fields]) OR "dental pulp capping"[All Fields] OR ("pulp"[All Fields] AND "capping"[All Fields]) OR "pulp capping"[All Fields]) OR "pulpotomy"[MeSH Terms] OR "pulp capping and pulpectomy agents"[MeSH Terms] | 5,879 | 23:59:36 |

**Supplementary S2.** List of potentially relevant studies not included in the systematic review, along with the reasons for exclusion.

| No. | Reference | Reason |
| --- | --- | --- |
| 1 | 1. Aguilar P, Linsuwanont P. Vital pulp therapy in vital permanent teeth with cariously exposed pulp: a systematic review. J Endod. 2011;37(5):581-587. doi:10.1016/j.joen.2010.12.004 2. Alqaderi HE, Al-Mutawa SA, Qudeimat MA. MTA pulpotomy as an alternative to root canal treatment in children's permanent teeth in a dental public health setting. J Dent. 2014;42(11):1390-1395. doi:10.1016/j.jdent.2014.06.007 3. Cushley S, Duncan HF, Lappin MJ, et al. Pulpotomy for mature carious teeth with symptoms of irreversible pulpitis: A systematic review. J Dent. 2019;88:103158. doi:10.1016/j.jdent.2019.06.005 4. Li Y, Sui B, Dahl C, et al. Pulpotomy for carious pulp exposures in permanent teeth: A systematic review and meta-analysis. J Dent. 2019;84:1-8. doi:10.1016/j.jdent.2019.03.010 | Review articles |
| 2 | 1. Odabaş ME, Alaçam A, Sillelioğlu H, Deveci C. Clinical and radiographic success rates of mineral trioxide aggregate and ferric sulphate pulpotomies performed by dental students. Eur J Paediatr Dent. 2012;13(2):118-122. | Pulpotomy on primary teeth |
| 3 | - 1. Azimi S, Fazlyab M, Sadri D, Saghiri MA, Khosravanifard B, Asgary S. Comparison of pulp response to mineral trioxide aggregate and a bioceramic paste in partial pulpotomy of sound human premolars: a randomized controlled trial. Int Endod J. 2014;47(9):873-881. doi:10.1111/iej.12231   2. Orhan AI, Oz FT, Orhan K. Pulp exposure occurrence and outcomes after 1- or 2-visit indirect pulp therapy vs complete caries removal in primary and permanent molars. Pediatr Dent. 2010;32(4):347-355.   3. Eghbal MJ, Asgary S, Baglue RA, Parirokh M, Ghoddusi J. MTA pulpotomy of human permanent molars with irreversible pulpitis. Aust Endod J. 2009;35(1):4-8. doi:10.1111/j.1747-4477.2009.00166.x | Studies not reporting on outcome of interest |
| 4 | 1. Özgür B, Uysal S, Güngör HC. Partial Pulpotomy in Immature Permanent Molars After Carious Exposures Using Different Hemorrhage Control and Capping Materials. Pediatr Dent. 2017;39(5):364-370. 2. Awawdeh L, Al-Qudah A, Hamouri H, Chakra RJ. Outcomes of Vital Pulp Therapy Using Mineral Trioxide Aggregate or Biodentine: A Prospective Randomized Clinical Trial. J Endod. 2018;44(11):1603-1609. doi:10.1016/j.joen.2018.08.004 3. Chailertvanitkul P, Paphangkorakit J, Sooksantisakoonchai N, et al. Randomized control trial comparing calcium hydroxide and mineral trioxide aggregate for partial pulpotomies in cariously exposed pulps of permanent molars. Int Endod J. 2014;47(9):835-842. doi:10.1111/iej.12225 4. Barngkgei IH, Halboub ES, Alboni RS. Pulpotomy of symptomatic permanent teeth with carious exposure using mineral trioxide aggregate. Iran Endod J. 2013;8(2):65-68. 5. Galani M, Tewari S, Sangwan P, Mittal S, Kumar V, Duhan J. Comparative Evaluation of Postoperative Pain and Success Rate after Pulpotomy and Root Canal Treatment in Cariously Exposed Mature Permanent Molars: A Randomized Controlled Trial. J Endod. 2017;43(12):1953-1962. doi:10.1016/j.joen.2017.08.007 6. Barrieshi-Nusair KM, Qudeimat MA. A prospective clinical study of mineral trioxide aggregate for partial pulpotomy in cariously exposed permanent teeth. J Endod. 2006;32(8):731-735. doi:10.1016/j.joen.2005.12.008 7. Keswani D, Pandey RK, Ansari A, Gupta S. Comparative evaluation of platelet-rich fibrin and mineral trioxide aggregate as pulpotomy agents in permanent teeth with incomplete root development: a randomized controlled trial. J Endod. 2014;40(5):599-605. doi:10.1016/j.joen.2014.01.009 8. Eppa HR, Puppala R, Kethineni B, Banavath S, Kanumuri PK, Kishore GVS. Comparative Evaluation of Three Different Materials: Mineral Trioxide Aggregate, Triple Antibiotic Paste, and Abscess Remedy on Apical Development of Vital Young Permanent Teeth. *Contemp Clin Dent*. 2018;9(2):158-163. doi:10.4103/ccd.ccd_587_17 | Diagnosis of reversible pulpitis/ unclear diagnostic data |
| 5 | 1. Taha NA, Ahmad MB, Ghanim A. Assessment of Mineral Trioxide Aggregate pulpotomy in mature permanent teeth with carious exposures. *Int Endod J*. 2017;50(2):117-125. doi:10.1111/iej.12605 2. Asgary S, Hassanizadeh R, Torabzadeh H, Eghbal MJ. Treatment Outcomes of 4 Vital Pulp Therapies in Mature Molars. *J Endod*. 2018;44(4):529-535. doi:10.1016/j.joen.2017.12.010 | Raw data not available to perform analysis on outcome of interest |
| 6 | 1. Asgary S, Eghbal MJ. Treatment outcomes of pulpotomy in permanent molars with irreversible pulpitis using biomaterials: a multi-center randomized controlled trial. *Acta Odontol Scand*. 2013;71(1):130-136. doi:10.3109/00016357.2011.654251 2. Asgary S, Eghbal MJ, Ghoddusi J, Yazdani S. One-year results of vital pulp therapy in permanent molars with irreversible pulpitis: an ongoing multicenter, randomized, non-inferiority clinical trial. *Clin Oral Investig*. 2013;17(2):431-439. doi:10.1007/s00784-012-0712-6 3. Asgary S, Eghbal MJ, Ghoddusi J. Two-year results of vital pulp therapy in permanent molars with irreversible pulpitis: an ongoing multicenter randomized clinical trial. *Clin Oral Investig*. 2014;18(2):635-641. doi:10.1007/s00784-013-1003-6 | Studies with duplication of data/ Follow up studies |
| 7 | 1. Dong Y, Hu SL, Sun YY, Zhang HY. *Shanghai Kou Qiang Yi Xue*. 2017;26(4):425-428. | Non-english language/ Full text english translation not available |

**Supplementary S3:** Modified Down and Black’s checklist for quality assessment of observational studies. The checklist includes the following domains: reporting, external validity, internal validity (bias), internal validity (confounding) and power. Downs and Black score ranges were classified as: excellent (26–28); good (20–25); fair (15–19); and poor (≤14).

|  |  | **Reporting** | **External validity** | **Internal validity** | **Confounding** | **Power** | **Total Score** |
| --- | --- | --- | --- | --- | --- | --- | --- |
|  |  | (11) | (3) | (7) | (6) | (1) | **(28)** |
| Study, year | Caliskan, 1993 [31] | 9 | 1 | 6 | 2 | 0 | **18** |
|  | Caliskan, 1995 [30] | 9 | 1 | 6 | 2 | 0 | **18** |
|  | Taha, 2018 [23] | 10 | 2 | 4 | 2 | 0 | **18** |
|  | Taha, 2018 [24] | 10 | 2 | 4 | 3 | 0 | **18** |
|  | Linsuwanont , 2017 [7] | 11 | 2 | 4 | 4 | 0 | **19** |
|  | Qudeimat, 2017 [26] | 10 | 2 | 4 | 2 | 0 | **18** |

**Supplementary S4:** Risk of bias assessment in randomized control trial included in the study using Cochrane risk of bias assessment 2 (ROB 2) tool.


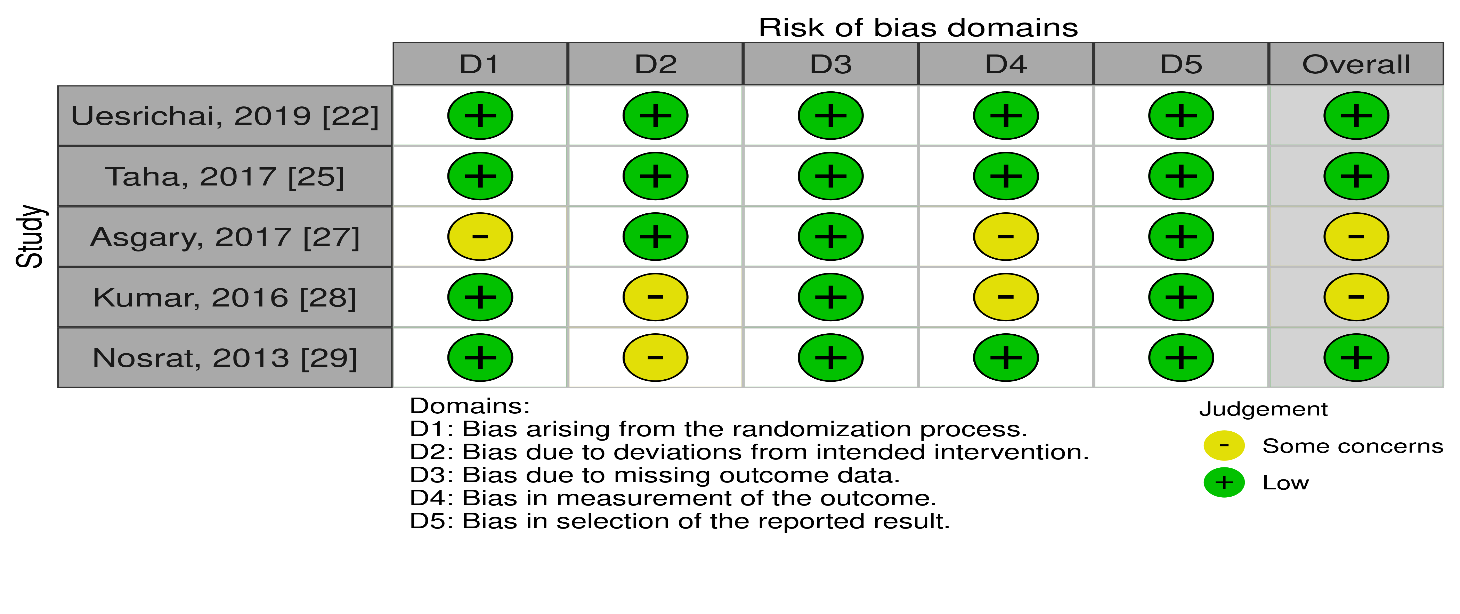

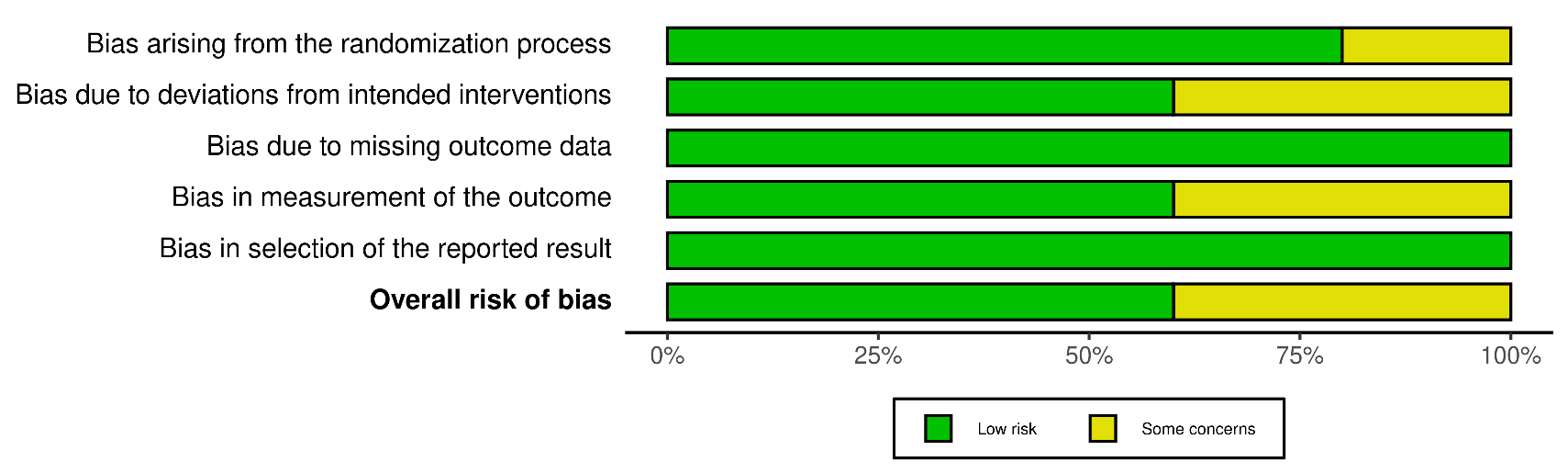


**Supplementary S5:** Indirect comparison of pulpotomy success rate based on different pulp capping materials. Comparison performed using z statistical test for two proportions.

|  | **z statistic** | **p-value** |
| --- | --- | --- |
| **MTA** vs **Ca(OH)2** | 2.18 | 0.03 |
| **MTA** vs **BD** | -3.39 | 0.0007 |
| **MTA** vs **CEM** | 1.5 | 0.13 |
| **BD** vs **Ca(OH)2** | 4.63 | 0 |
| **BD** vs **CEM** | 4.2 | 0 |
| **Ca(OH)2** vs **CEM** | 0.85 | 0.4 |

**Supplementary S6:** Funnel plot showing asymmetric distribution of studies indicating possible publication bias


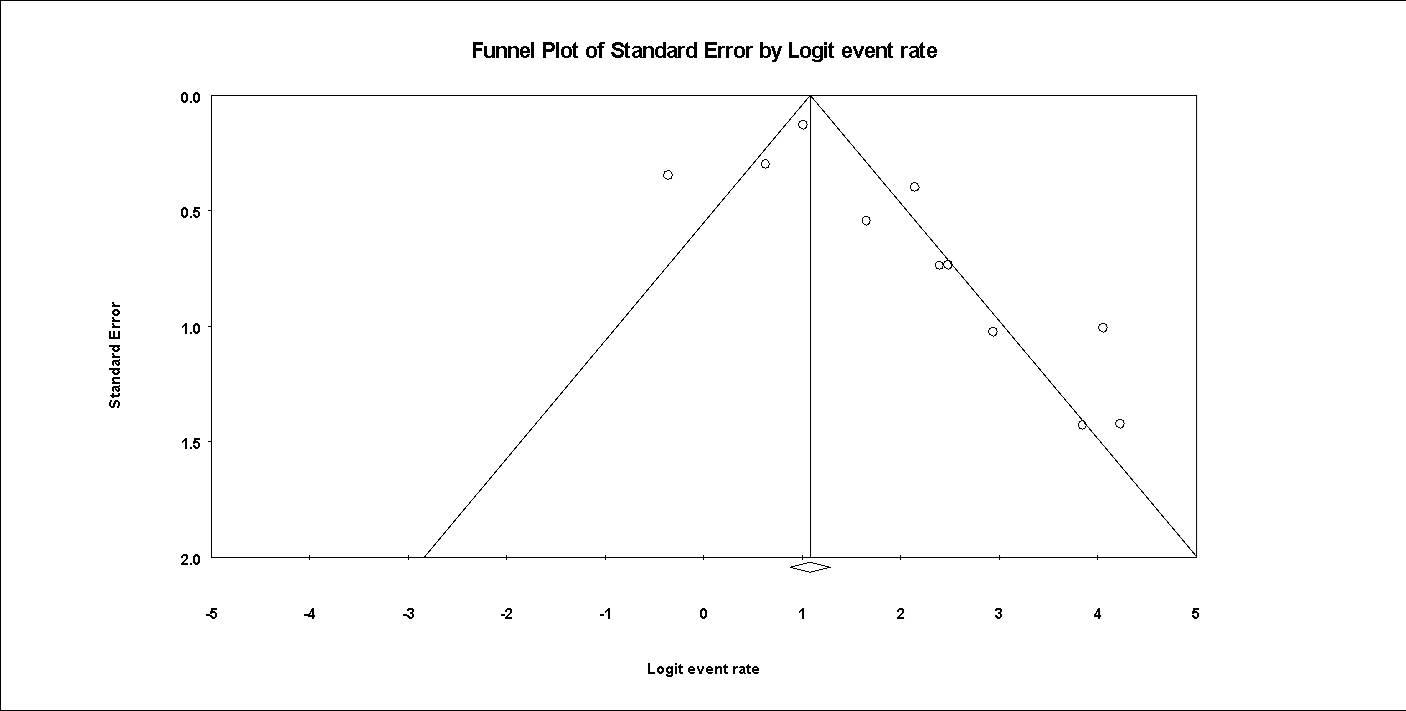

Supplement: Supplementary file 1 — Supplementary Information 1. [file 41598_2022_20918_MOESM1_ESM.docx]
